# The genome sequence of *Molossus* *nigricans* (Chiroptera, Molossidae; Miller, 1902)

**DOI:** 10.12688/wellcomeopenres.18724.1

**Published:** 2023-05-05

**Authors:** Nancy B. Simmons, Melissa R. Ingala, Myrtani Pieri, Thomas L. Volkert, Larry N. Singh, Philge Philip, Laramie L. Lindsey, Ning Zhang, Jonathan L. Gray, Brian P. O'Toole, Meike Mai, Emma C. Teeling, Sonja C. Vernes, the Bat Biology Foundation, the Bat1K Consortium

**Affiliations:** 1 Department of Mammalogy, Division of Vertebrate Zoology, American Museum of Natural History, New York, NY10024, USA; 2 National Museum of Natural History, Smithsonian Institution, Washington, DC 20560, USA; 3 Department of Biological Sciences, Fairleigh Dickinson University, Madison, NJ 07940, USA; 4 Department of Life Sciences, School of Life and Health Sciences, University of Nicosia, Nicosia, Cyprus; 5 Paratus Sciences, New York NY, USA; 6 School of Biology, The University of St Andrews, St Andrews, UK; 7 School of Biology and Environmental Science, University College Dublin, Dublin, Ireland; 8 Wellcome Sanger Institute, Wellcome Genome Campus, Cambridgeshire, CB10 1SA, UK; 9 Neurogenetics of Vocal Communication Group, Max Planck Institute for Psycholinguistics, Nijmegen, The Netherlands

**Keywords:** Molossus nigricans, genome sequence, chromosomal, Bat1K

## Abstract

We present a genome assembly from an individual male *Molossus* *nigricans* (Chordata; Mammalia; Chiroptera; Molossidae). The genome sequence is 2.41 gigabases in span. The majority of the assembly is scaffolded into 24 chromosomal pseudomolecules, with the X sex chromosome assembled.

## Species taxonomy

Eukaryota; Metazoa; Chordata; Craniata; Vertebrata; Euteleostomi; Mammalia; Eutheria; Laurasiatheria; Chiroptera; Yangochiroptera; Vespertilionoidea, Molossidae; Molossinae; *Molossus*; *Molossus nigricans,* (Meredith et al Science 2011; Teeling *et al.* Science 2005; Miller, 1902) 

## Introduction

**Figure 1.**
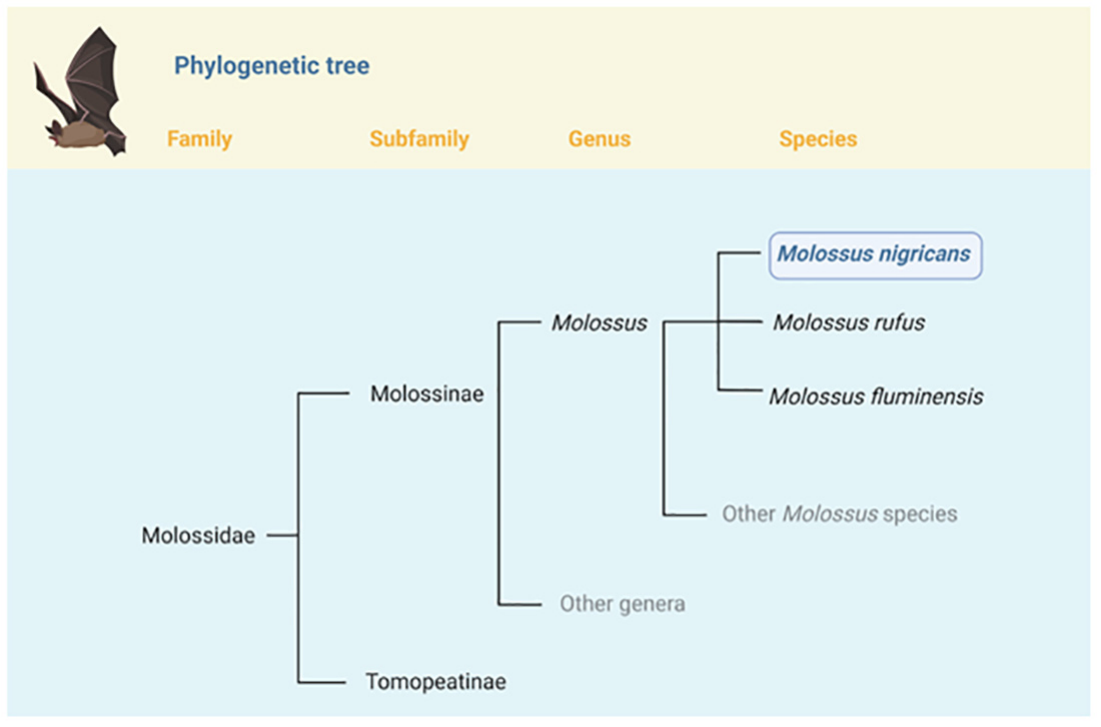
Position of *Molossus nigricans* in the phylogeny of Family Molossidae. *Molossus nigricans* is one of 15 species currently recognized in the genus *Molossus* ^2– 4^. *Molossus* belongs to the Subfamily Molossinae, which currently includes 21 genera and 131 species ^2^. Within *Molossus*, the closest relatives of *M. nigricans* are *M. rufus* and *M. fluminensis;* until recently these taxa were considered conspecific ^4^.

**Figure 2.**
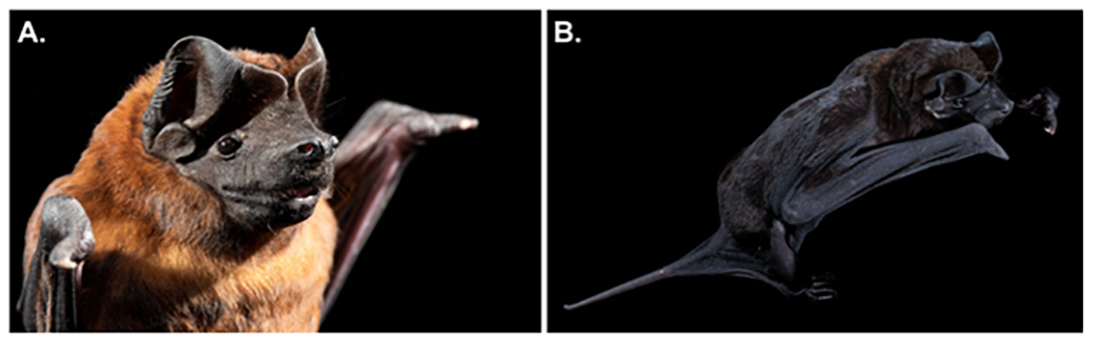
*Molossus nigricans* bats. Adult individuals of the *Molossus nigricans* bat come in two color morphs. A red adult individual shown in ( **A**) and an individual with black pelage in ( **B**) [Photos taken at Lamanai, Belize by Brock and Sherri Fenton].

Molossid bats are swift aerial insectivores that are distributed throughout the world. As shown in Figure 1, they comprise two subfamilies, the South American endemic Tomopeatinae and the cosmopolitan Molossinae ^1^, the latter consisting of 21 genera and 131 species ^2^. Within this group, the genus *Molossus* comprises 15 species distributed broadly across the Neotropics ^2, 3^. *Molossus nigricans*, one of the largest species of *Molossus*, is found in Central America from southern Mexico, Belize, and Guatemala to Panama ^2, 4– 6^. Adults of this species come in one of two color morphs, either black or red; a red individual is shown in Figure 2A and an individual with black pelage is shown in Figure 2B. Until recently *M. nigricans* was considered to be a subspecies of *M. rufus* ^7^, but Loureiro *et al.* ^3, 4^ demonstrated that it represents a distinct species.

The echolocation calls of *M*. *nigricans* are frequency modulated (FM) in the short first step of the call while the rest of the call is constant frequency (CF) ^8^. The 20–35 kHz frequency range for molossid bats is apparently related to foraging strategies, allowing these species greater success in their open space foraging habitats ^9^. Typically, molossid bats feed in open areas at relatively high altitudes where their relatively low-frequency echolocation calls maximize response times and allow for better detection of a wide range of potential prey of different sizes ^9, 10^. In Belize, *M. nigricans* has a diverse diet including mostly beetles and hemipterans ^11^. The species *M*. *nigricans* is not currently classified in the IUCN Red List of Threatened Species; the species complex from which it was split, *Molossus rufus,* is classified as Least Concern ^12^.

The genus *Molossus* is morphologically conservative, and the level of genetic divergence is also low among species, which has masked recognition of the actual species diversity in the genus. Recently Loureiro *et al.* ^3, 4^ clarified the taxonomy of *Molossus* and increased the number of recognized species by nearly 50%. Notably, they found that the wide-spread taxon formerly known as *Molossus rufus* is actually a complex of cryptic species ^4^. Loureiro *et al.* (2020), subsequently divided *M*. *rufus* into three species, validating old names previously considered synonyms/subspecies of *M*. *rufus*: *Molossus rufus* E. Geoffroy, 1805, *Molossus nigricans Miller 1902* and *Molossus fluminensis* Lataste, 1891 ( Figure 1).

### Genome sequence report

The genome was sequenced from a single male *M*. *nigricans* (field number BZ-404, catalog number AMNH:Mammalogy:280920) collected from the Lamanai Archaeological Reserve, Orange Walk District, Belize on 12 November 2021. A total of 41-fold coverage in Pacific Biosciences Hi-Fi long reads (contig N50 21 Mb) was generated after removal of all reads shorter than 10kb. Primary assembly contigs were scaffolded with chromosome confirmation Hi-C data. The final assembly has a total length of 2.41 Gb in 146 sequence scaffolds with a scaffold N50 of 81.9 Mb ( Table 1). The majority, 79.45%, of the assembly sequence was assigned to 24 chromosomal-level scaffolds, representing 23 autosomes (numbered by sequence length, and the X sex chromosome). Chromosomal pseudomolecules in the genome assembly of *Molossus nigricans* are shown in Table 2. The assembly has a BUSCO ^13^ completeness of 96.1% using the laurasiatheria reference set. While not fully phased, the assembly deposited is of one haplotype.

**Table 1.** Genome data for *Molossus nigricans*.

*Project accession data*	
Assembly identifier	mMolNig1
Species	*Molossus nigricans*
Specimen	mMolNig1
NCBI taxonomy ID	NCBI:txid2997257 Until recently *M. nigricans* was considered to be a subspecies of *M. rufus* (NCBI taxonomy ID of *M. rufus* is NCBI:txid124751)
BioProject	PRJNA489245
BioSample ID	SAMN31835895
Isolate information	Male - Muscle
Genome assembly	
Assembly accession	GCA_026936385.1
Bioproject for Assembly	PRJNA904257
WGS accession for Assembly	JAPNNZ000000000
Span (Mb)	2,407.89
Number of contigs	345
Contig N50 length (Mb)	21.89
Number of scaffolds	146
Scaffold N50 length (Mb)	81.93
Longest scaffold (Mb)	241.11

* BUSCO scores based on the mammalia_odb10 BUSCO set using v5.0.0. C= complete [S= single copy, D=duplicated], F=fragmented, M=missing, n=number of orthologues in comparison.

* *Molossus nigricans* BUSCO scores based on laurasiatheria_odb10 BUSCO set v5.3.2.

**Table 2.** Chromosomal pseudomolecules in the genome assembly of *Molossus* *nigricans*. ENA accession Chromosome Size (Mb) and GC%. The chromosome number of *Molossus nigricans* is 2n=48.

ENA accession	Chromosome	Size (Mb)	GC%
Scaffold_1	1	241.11	40.62
Scaffold_2	2	119.36	42.67
Scaffold_3	3	109.88	39.90
Scaffold_4	4	108.50	39.75
Scaffold_5	5	93.61	39.89
Scaffold_6	6	92.09	42.63
Scaffold_7	7	91.92	39.97
Scaffold_8	8	91.72	39.62
Scaffold_9	9	87.41	39.43
Scaffold_10	10	87.24	40.84
Scaffold_11	11	81.93	43.27
Scaffold_12	12	81.17	41.86
Scaffold_13	13	80.48	41.73
Scaffold_14	14	72.26	43.77
Scaffold_15	X	69.55	38.52
Scaffold_16	15	62.01	43.64
Scaffold_17	16	61.83	42.23
Scaffold_18	17	61.70	44.01
Scaffold_19	18	50.87	45.85
Scaffold_20	19	48.85	41.04
Scaffold_21	20	38.31	47.15
Scaffold_22	21	30.68	39.55
Scaffold_23	22	25.72	44.94
Scaffold_24	23	24.72	42.99

## Methods

The *M*. *nigricans* specimen was a male individual of black pelage collected on an American Museum of Natural History (AMNH) field expedition at the Lamanai Archaeological Reserve in the Orange Walk District of Belize. The individual sampled was identified as *M. nigricans* based on morphometrics ( *e.g.*, forearm length, body mass) and morphological traits ( *e.g.*, fur color pattern) described by Loureiro *et al.* ^4^.” The bat was caught in a 30 x 100 ft macro mist net ^14^ set in the High Temple Plaza at Lamanai (17.76736 N, 88.65270 W), an area known to be near a roost of this species. All efforts were made to minimize any distress or suffering by the animal. The individual sampled was subjected to minimal handling after capture, and it was held in a clean cloth bag after capture as per best practices for field containment of bats. After species identification, the individual was euthanized humanely the same night it was captured. The animal was identified as *M. nigricans* based on morphometrics and morphological traits described by Loureiro *et al.* ^4^. The animal was euthanized by isoflurane inhalation, a humane approved method that rapidly causes unconsciousness and eventually death upon inhalation. Bats euthanized by this method are rendered unconscious within seconds due to their high respiration rate, and death occurs within a minute or two with no significant suffering by the animal. Capture and sampling were conducted under Belize Forest Department Permit FD/WL/1/21(16) and Belize Institute of Archaeology Permit IA/S/5/6/21(01), and samples were exported under Belize Forest Department permit FD/WL/7/22(08). All work was conducted with approval by the AMNH Institutional Animal Care and Use Committee (AMNHIACUC-20191212) ^15^. All data were recorded and reported in accordance with the ARRIVE guidelines ^16^ – see data availability section and Table 1. Tissues were removed from the subject individual immediately following euthanasia and were flash-frozen in a liquid nitrogen dry shipper, with the cold chain maintained from field to museum to laboratory. DNA was extracted using Nanobind extraction from muscle tissue following the Circulomics Nanobind HMW DNA Extraction Protocol. Pacific Biosciences HiFi libraries were constructed according to the manufacturer's instructions. Hi-C data was generated using the Arima Hi-C+ High Coverage kit from the same muscle tissue sample. Sequencing was performed by the Genomic Operations DNA Pipelines at Paratus Sciences on Pacific Biosciences Sequel IIe (HiFi reads) and Illumina NextSeq 2000 (Hi-C) instruments.

Assembly was carried out following the Vertebrate Genome Project pipeline v2.0 ^17^ with a few modifications as follows. An initial QC analysis was performed on the raw BAM file using FastQC. BAM files were converted to fastq format and merged for downstream processing. Genome size was estimated using GenomeScope2 ^18^. HiCanu was used for genome assembly ^19^. Haplotypic duplication was identified and removed with purge dups ^20^. The quality of the assembly was evaluated using Merqury ^17^ and BUSCO ^21^. Scaffolding with Hi-C data ^22^ was carried out with SALSA2 ^23^ HiGlass ^24^ was implemented to generate Hi-C contact maps. Figure 2– Figure 6 were generated using BlobToolKit ^25^. Software utilised for the *Molossus nigricans* genome analyses are depicted in Table 3.

**Figure 3.**
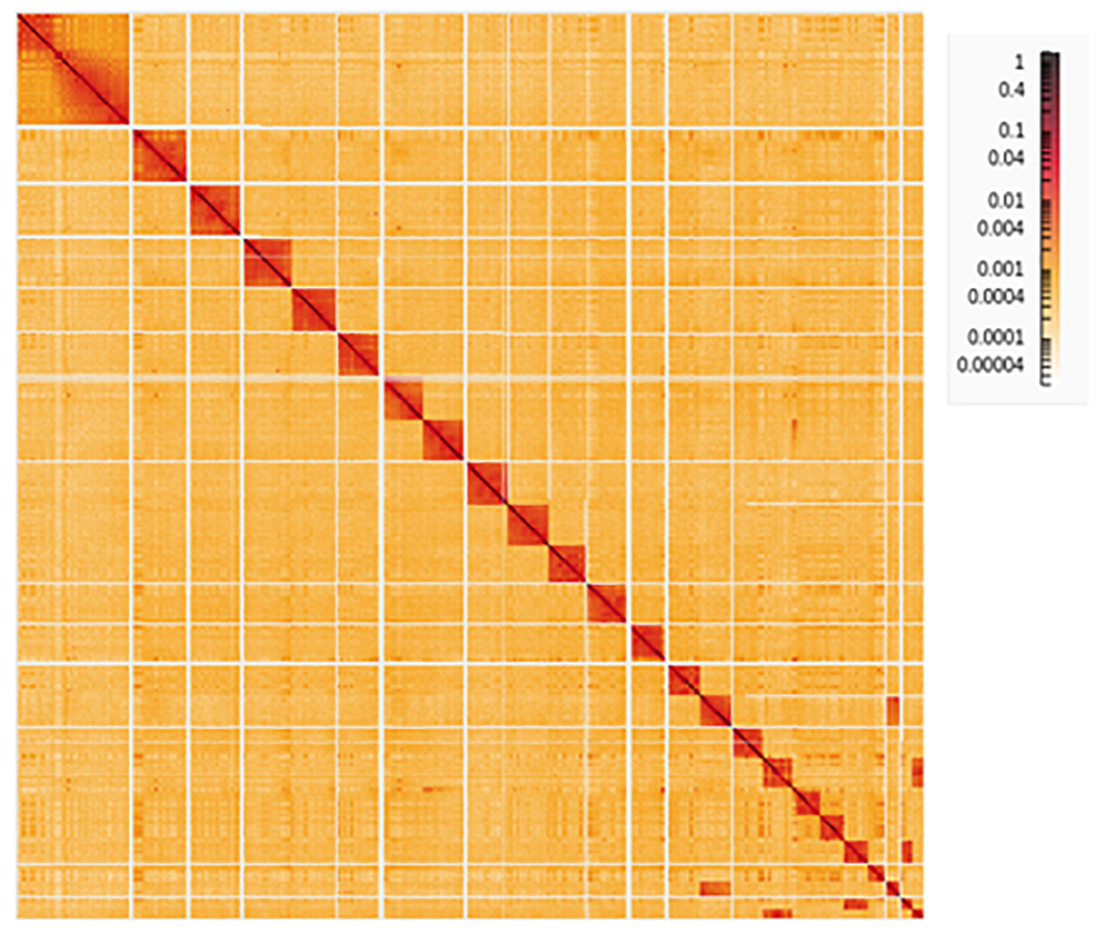
Hi-C Contact Map of the *Molossus nigricans* assembly with 24 chromosomes, visualized using HiGlass.

**Figure 4.**
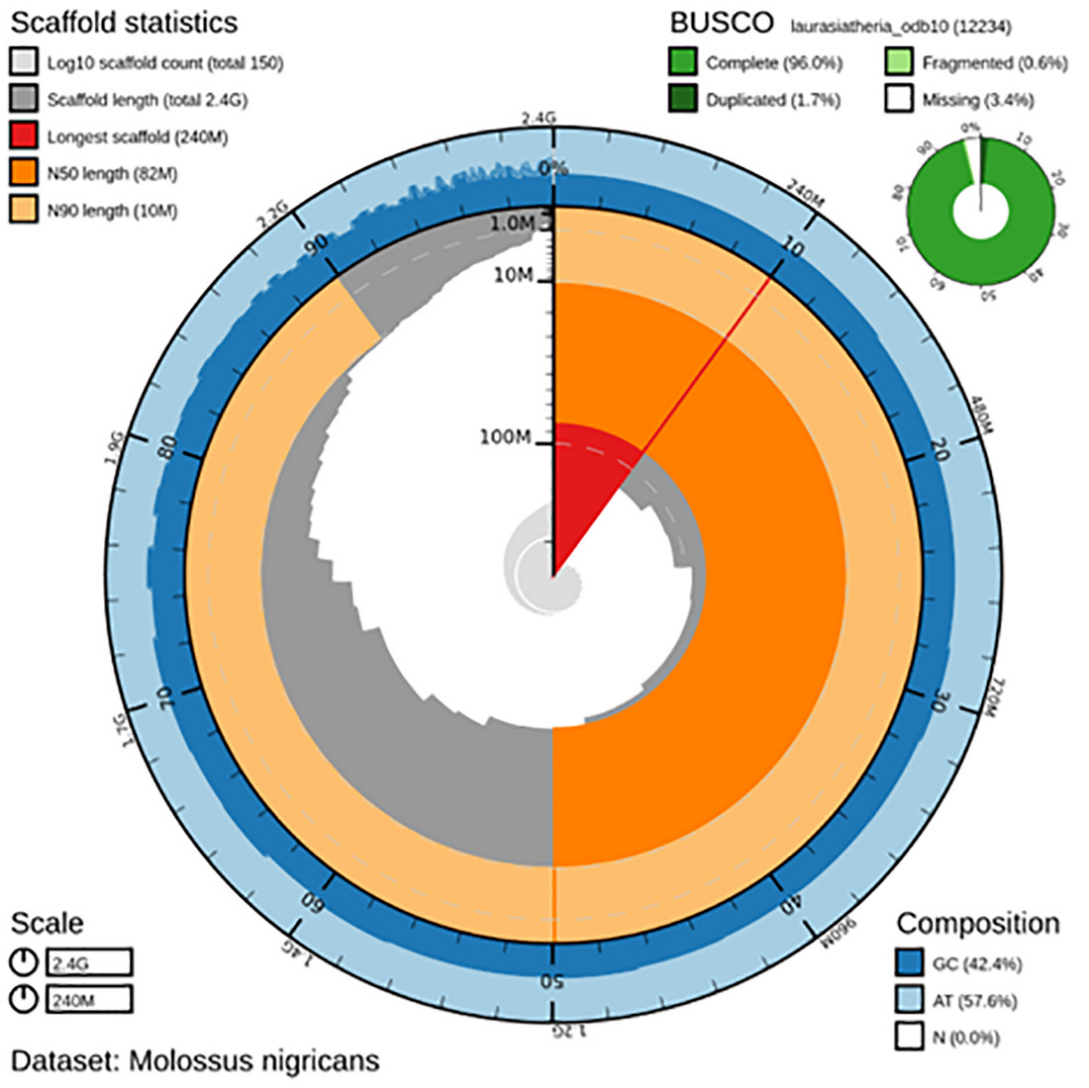
Genome assembly metrics generated using blobtoolkit for the *Molossus nigricans* genome assembly. The larger snail plot depicts scaffold statistics including N50 length (bright orange) and base composition (blue). The smaller plot shows BUSCO completeness in green.

**Figure 5.**
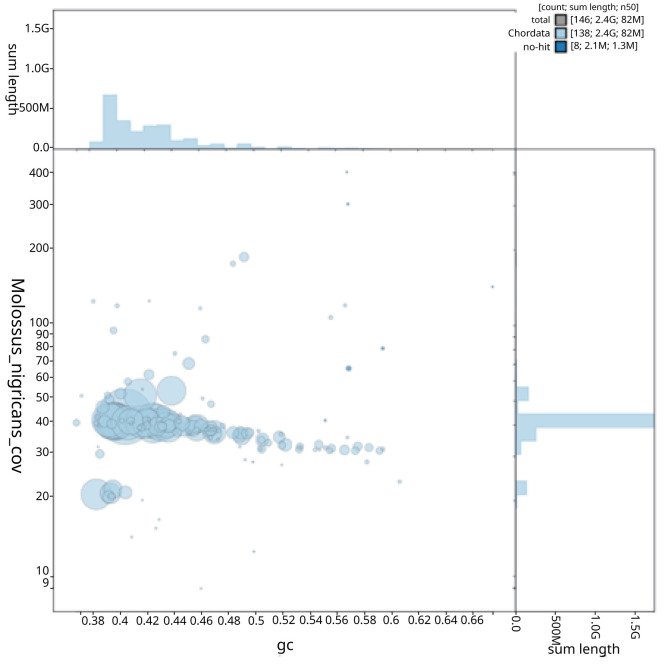
GC coverage plot generated for the *Molossus nigricans* assembly using blobtoolkit. Individual chromosomes and scaffolds are represented by each circle. The circles are sized in proportion to chromosome/scaffold length. Histograms show the sum length of chromosome/scaffold size along each axis. Color of circles indicate taxonomic hits of each Phylum represented in the assembly.

**Figure 6.**
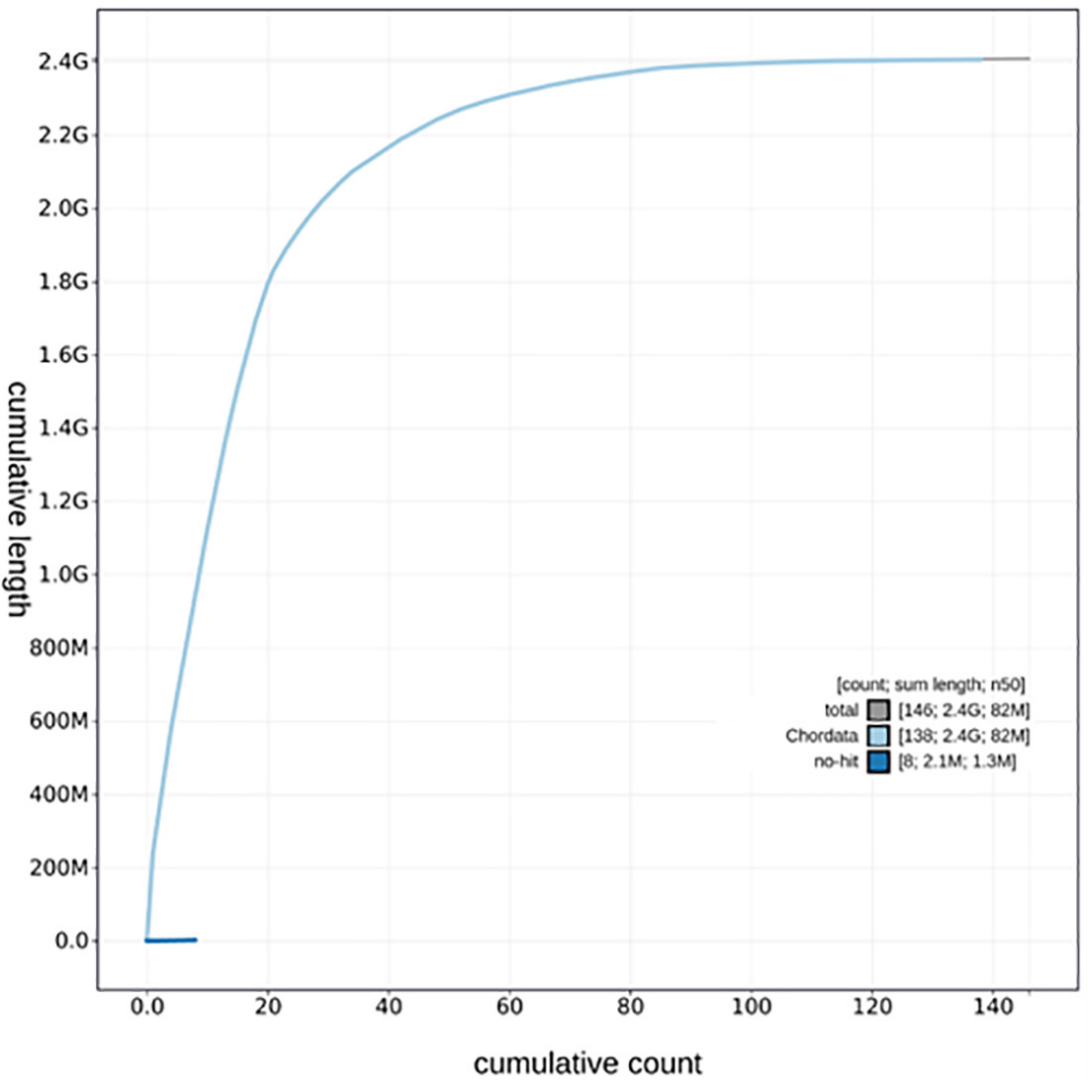
Cumulative sequence plot generated for the Molossus nigricans assembly using blobtoolkit. The grey line shows the cumulative length for all chromosomes/scaffolds in the assembly. Colored lines represent Phylum represented in the assembly.

**Table 3.** Software tools used.

Software tool	Version	Source
bamUtil	1.0.15	https://genome.sph.umich.edu/wiki/BamUtil:_bam2FastQ
FastQC	0.11.9	https://www.bioinformatics.babraham.ac.uk/projects/fastqc/
MultiQC	1.13	https://github.com/ewels/MultiQC
Genomescope	2.0	https://github.com/tbenavi1/genomescope2.0
HiCanu	2.2	https://github.com/marbl/canu
purge_dups	1.2.6	https://github.com/dfguan/purge_dups
BUSCO	5.3.2	https://busco.ezlab.org/
Merqury	1.3	https://github.com/marbl/merqury
Assembly-stats	17.02	https://github.com/rjchallis/assembly-stats
Arima-HiC Mapping Pipeline	-	https://github.com/ArimaGenomics/mapping_pipeline
SALSA	2	https://github.com/marbl/SALSA
HiGlass	1.11.7	https://github.com/higlass/higlass
samtools	1.9	https://www.htslib.org/
BlobToolKit	3.2.7	https://github.com/blobtoolkit/blobtoolkit

## Data Availability

The *M. nigricans* genome sequencing initiative is part of the Bat1K genome sequencing project. The genome assembly is released openly for reuse. Underlining data may be available for non-commercial research purposes upon request. Please email info@bbf.org for more information. The genome assembly can be found in the European Nucleotide Archive: *Molossus nigricans* (northern black mastiff bat). Accession number: GCA_026936385.1, https://identifiers.org/insdc.gca:GCA_026936385.1 ^26^ NCBI BioProject: Molossus nigricans isolate: mMolNig1 (northern black mastiff bat). Accession number: PRJNA904257, https://identifiers.org/ncbiprotein:PRJNA904257 ^27^ under the Bat1K BioProject PRJNA489245. Data accession identifiers are reported in Table 1.
