# Supplementary material for: The genome sequence of Molossus nigricans (Chiroptera, Molossidae; Miller, 1902)
Source: Wellcome Open Res. 2023 May 5;8:198. [Version 1] doi: 10.12688/wellcomeopenres.18724.1 (PMC10435916; doi:10.12688/wellcomeopenres.18724.1)
Supplement: Peer review history

# Peer review: Reviewer response for version 1

**DOI:** 10.21956/wellcomeopenres.20762.r62477

**Published:** 2023-08-17

**Authors:** Linelle Ann Lacson Abueg

**Affiliations:** 1 The Vertebrate Genome Laboratory, The Rockefeller University, New York, NY, USA

The authors provide an appropriate and well-detailed description of their new M. nigricans genome. It might be interesting to note if other bats from the M. rufus species complex are also in the sampling area where this individual was found. Nonetheless, this genome will be a useful resource for those studying the genomics of molossid bats. 

 Comments:

- in Genome Sequence Report: "While not fully phased, the assembly deposited is of one haplotype." I don't think the "one haplotype" part can be asserted here given that it is a pseudohaplotype assembly. Haplotype switches are still possible.
- Table 2: is there a citation (karyotype?) for the chromosome number for M. nigricans mentioned in the caption, or is this referring to the observed scaffolds?
- in Methods, these sentences: "The animal was identified as [...] within a minute or two with no significant suffering by the animal." are just re-stating the previous sentences starting with "The individual sampled was identified as [...] humanely the same night it was captured." but with more detail about the euthanasia. Maybe keep the second set of sentences, adding in the mist net information from the first set.
- in Methods: "Vertebrate Genome Project" should be "Vertebrate Genomes Project". Spell out "QC" before using acronym. There is a period missing between "Scaffolding with Hi-C data" part and "HiGlass" part.
- in Methods: the HiGlass image/legend and snail plot are blurry upon downloading? GC and cum. seq. plots are fine.
- in Data Availability: "Underlining data" should be "underlying data"

Are sufficient details of methods and materials provided to allow replication by others?

Yes

Is the rationale for creating the dataset(s) clearly described?

Yes

Are the datasets clearly presented in a useable and accessible format?

Yes

Are the protocols appropriate and is the work technically sound?

Yes

Reviewer Expertise:

Genome assembly

I confirm that I have read this submission and believe that I have an appropriate level of expertise to confirm that it is of an acceptable scientific standard.

# Peer review: Reviewer response for version 1

**DOI:** 10.21956/wellcomeopenres.20762.r62476

**Published:** 2023-08-10

**Authors:** Jamie McGowan

**Affiliations:** 1 Earlham Institute, Norwich, UK

Simmons *et al. *report a reference quality genome assembly for *Molossus nigricans*, generated from an individual male bat. The assembly was constructed by assembling PacBio HiFi reads using HiCanu followed by scaffolding with Hi-C data using SALSA2. Overall, the data note is well written and the methods are described well. The genome assembly is high quality both in terms of contiguity and predicted completeness. The assembly has already been publicly deposited.

 I have one concern. How confident are the authors that *M. nigricans* has 24 chromosomes? From the Hi-C contact map in Figure 3, it looks like the last three “chromosomes” on the bottom right have significant contact with three larger “chromosomes”. I wondered if these were missed joins that should have been scaffolded together? If this were the case, it would reduce the number of chromosomes. Have the authors attempted manually curating the assembly to validate the number of chromosomes?

**Minor comments**

1. The caption for Table 1 includes two asterisks notes regarding BUSCO but the table does not include the BUSCO results.
2. There is some repeated text in the methods section “…was identified as *M. nigricans* based on morphometrics….”.

Are sufficient details of methods and materials provided to allow replication by others?

Yes

Is the rationale for creating the dataset(s) clearly described?

Yes

Are the datasets clearly presented in a useable and accessible format?

Yes

Are the protocols appropriate and is the work technically sound?

Yes

Reviewer Expertise:

Genome assembly, evolutionary genomics

I confirm that I have read this submission and believe that I have an appropriate level of expertise to confirm that it is of an acceptable scientific standard, however I have significant reservations, as outlined above.

# Peer review: Reviewer response for version 1

**DOI:** 10.21956/wellcomeopenres.20762.r56986

**Published:** 2023-07-10

**Authors:** Vincent J. Lynch

**Affiliations:** 1 Department of Biological Sciences, University at Buffalo, Buffalo, New York, USA

Simmons et al. report the chromosome-level genome of a male Northern Black Mastiff Bat ( *Molossus nigricans*) generated with Pacific Biosciences Hi-Fi long reads. The genome quality looks excellent and methods well described; the sequencing data and genome assembly are already publicly available. I see no issues that need to be addressed before indexing.

Are sufficient details of methods and materials provided to allow replication by others?

Yes

Is the rationale for creating the dataset(s) clearly described?

Yes

Are the datasets clearly presented in a useable and accessible format?

Yes

Are the protocols appropriate and is the work technically sound?

Yes

Reviewer Expertise:

Evolutionary biology, DevoEvo, genetics, genomics, and molecular evolution

I confirm that I have read this submission and believe that I have an appropriate level of expertise to confirm that it is of an acceptable scientific standard.
